# Revisiting Species Distribution and Antifungal Susceptibility of *Candida* Bloodstream Isolates from Latin American Medical Centers

**DOI:** 10.3390/jof3020024

**Published:** 2017-05-17

**Authors:** Daniel Archimedes da Matta, Ana Carolina Remondi Souza, Arnaldo Lopes Colombo

**Affiliations:** Laboratório Especial de Micologia, Disciplina de Infectologia, Escola Paulista de Medicina, Universidade Federal de São Paulo, São Paulo, SP 04039-032, Brazil; darchimedes@hotmail.com (D.A.d.M.); carolina.remondi@yahoo.com.br (A.C.R.S.)

**Keywords:** candidemia, *Candida* spp., nosocomial infection, infections acquired in a healthcare setting, antifungal resistance, opportunistic infections

## Abstract

The epidemiology of candidemia varies geographically, and there is still scarce data on the epidemiology of candidemia in Latin America (LA). After extensive revision of medical literature, we found reliable and robust information on the microbiological aspects of candidemia in patients from 11 out of 21 medical centers from LA countries and 1 out of 20 from Caribbean countries/territories. Based on 40 papers attending our search strategy, we noted that *C. albicans* remains the most common species causing candidemia in our region, followed by *C. parapsilosis* and *C. tropicalis*. In Argentina, Brazil, and Colombia, a trend towards an increase in frequency of *C. glabrata* candidemia was observed. Although resistance rates to fluconazole is under 3%, there was a slight increase in the resistance rates to *C. albicans*, *C. parapsilosis* and *C. tropicalis* isolates. Echinocandin resistance has been reported in a few surveys, but no single study confirmed the resistant phenotype reported by using molecular methods. We highlight the importance of conducting continuous surveillance studies to identify new trends in terms of species distribution of *Candida* and antifungal resistance related to episodes of candidemia in LA. This information is critical for helping clinicians to prevent and control *Candida* bloodstream infections in their medical centers.

## 1. Introduction

Fungi has been increasingly recognized as major agents of nosocomial infections in contemporary medicine, with emphasis on *Candida* spp., that accounts for the majority of invasive fungal infections associated in healthcare facilities. Indeed, besides being highly prevalent in Latin America countries, candidemia is associated with high morbidity and mortality rates, especially in critically ill patients [1,2,3,4,5,6,7].

The epidemiology of candidemia varies geographically, and, although *C. albicans* is still considered the most common cause of candidemia, there is a concern with the increasing rates of invasive infections due to *Candida* non-*albicans* (CNA) species in different parts of the world [7,8,9,10]. The reasons for the differences in species distribution and for the emergence of CNA species are not fully understood. However, there seems to be an association with a combination of variables related to host underlying conditions and medical interventions, including different practices of antifungal prophylaxis and resources available for prevention of health care associated infections [5,9,11,12,13,14,15,16,17].

Recognition of the importance of *Candida* infections has led to a significant increase in the use of antifungal agents in regiments of prophylaxis and empirical therapy, resulting in the emergence of resistant clinical isolates, particularly against triazoles and echinocandins [18,19]. In this scenario, the recent emergence of fluconazole resistance among isolates that are usually primarily sensitive to this drug, including *C. parapsilosis* and *C. tropicalis* strains, is worth mentioning [8,20,21,22,23,24]. Regarding to echinocandins, though resistance is still considered uncommon, occurrence of isolates with lower susceptibility to this therapeutic class has been increasingly reported, particularly among *C. glabrata* isolates [19,25,26,27].

The awareness of local epidemiology and antifungal susceptibility profile of *Candida* infections in different medical centers has major relevance in terms of guiding clinicians in optimizing their strategy for prevention and treatment of such fungal infections. This review attempts to provide a better understanding of the landscape of species distribution and antifungal resistance rates among *Candida* bloodstream isolates obtained from patients admitted in Latin American and Caribbean medical centers.

## 2. Methods

Our search strategy for the literature review was based on extensive revision of publications listed at PubMed, Scientific Electronic Library Online (SciELO), and MEDLINE bibliographic databases along the period between 1997 and 2016, irrespective of language and country of publication. Papers were retrieved using the following key words: candidemia, fungemia, *Candida* bloodstream infection, invasive candidiasis, *Candida* species distribution, antifungal susceptibility, and antifungal resistance. For each of these subjects, we also narrowed our search by adding the continent and country including the following: Central America, Latin America, South America, Caribbean, Argentina, Bolivia, Brazil, Chile, Colombia, Costa Rica, Cuba, Dominican Republic, Haiti, Honduras, Jamaica, Mexico, Panama, Peru, Puerto Rico, Uruguay, and Venezuela. Papers were included in our analysis only if their methodology and results matched the following criteria: (i) *Candida* isolates were identified at species level by a reliable and accurate phenotypic or molecular method; (ii) availability of data related to at least 50 *Candida* bloodstream isolates sequentially collected from different patients admitted in tertiary hospitals along the period of study; (iii) information regarding antifungal susceptibility of *Candida* spp. generated by The European Committee on Antimicrobial Susceptibility Testing (EUCAST) or Clinical Laboratory Standard Institute (CLSI) microbroth methods. Rates of resistance to triazoles and echinocandins reflect the breakpoints available at the time when the study was published. Papers addressing identification and susceptibility tests of worldwide collections of *Candida* isolates were considered only if isolates were obtained from patients with candidemia and data from Latin American or Caribbean countries were addressed separately. For antifungal susceptibility results, we only considered studies that tested at least 60% of all original collections of *Candida* strains originally selected for the study.

## 3. Results

We identified 40 studies that contained our inclusion criteria providing data of species distribution and antifungal susceptibility of *Candida* strains related to episodes of candidemia documented in Latin American and Caribbean medical centers.

**Figure 1:**
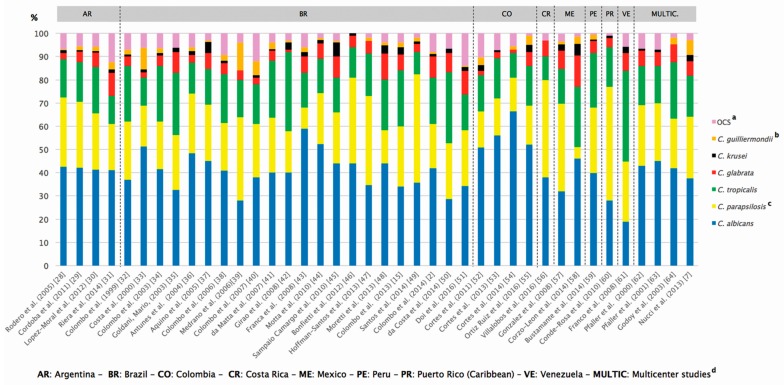
Proportion of the most common *Candida* species isolated from episodes of candidemia in Latin American and Caribbean medical centers (1997–2016). ^a^ Other Candida Species—species other than *C. albicans*. *C. parapsilosis* (sensu lato). *C. tropicalis*. *C. glabrata*. *C. krusei*. *C. guilliermondii* (sensu lato); ^b^ *C. guilliermondii* (sensu lato); ^c^ *C. parapsilosis* (sensu lato); ^d^ Multicenter studies including different countries from Latin America.

In terms of geographic origin, 22 papers were from Brazil, 4 from Argentina, 4 from Colombia, 2 from Mexico, and 1 each representing the following countries: Costa Rica, Peru, Venezuela, and Puerto Rico (Caribbean region). Of note, Puerto Rico is an unincorporated territory of the United States of America (USA), located in the Caribbean. In addition to 36 studies reporting data of single countries, we also evaluated results provided by 4 multicenter studies that tested *Candida* spp. bloodstream isolates from multiple Latin American countries including Argentina, Brazil, Chile, Colombia, Costa Rica, Ecuador, Honduras, Mexico, Peru, Uruguay, and Venezuela. In this scenario, we identified papers characterizing the etiologic pattern and antifungal susceptibility of episodes of candidemia documented in only 11 out of 21 Latin American countries and 1 out of 20 Caribbean countries and territories. Taking together all data available, we were able to characterize the species distribution in more than 12,000 episodes of *Candida* bloodstream infections documented in 40 medical centers from our region (Table 1, Figure 1, and Supplementary Material). Among all *Candida* spp. isolates analysed for species distribution, only 5460 isolates had their antifungal susceptibility profile performed in 17 studies (Table 2).

*C. albicans*, *C. parapsilosis* (sensu lato), *C. tropicalis*, and *C. glabrata* were the most commonly found species causing candidemia in all countries. *C. albicans* was the main cause of candidemia in 32 out of 40 studies exhibiting prevalence rates ranging between 18.8% and 66%. The highest prevalence rate of *C. albicans* candidemia was reported by Cortes et al. (2014) in Colombia [54]. It is worth to mention that all 4 studies from Colombia had *C. albicans* prevalence rates higher than 50%. In contrast, less than 30% of prevalence rates of *C. albicans* strains were reported in Brazil by Medrano et al. (2006) and da Costa et al. (2014), by Conde-Rosa et al. (2010) in a single study reported in Puerto Rico, and by Franco et al. (2008) in Venezuela [39,50,60,61]. In these studies, *C. tropicalis* and/or *C. parapsilosis* were the most commonly found species causing candidemia.

*C. parapsilosis* (sensu lato) isolates ranged from 5% to 49% of all candidemic episodes. Indeed, *C. parapsilosis* was identified as the main agent of CNA species candidemia in 25 out of 40 studies, and in 6 of the studies it surpassed the prevalence rate of *C. albicans*.

*C. tropicalis* was the second most common CNA species isolated in our region exhibiting prevalence rates ranging between 9.7% and 39%. Of note, *C. tropicalis* was the main cause of CNA species candidemia in 13 out of 40 studies. Franco et al. (2008) and da Costa et al. (2014) reported that *C. tropicalis* surpassed *C. albicans* in their surveys of candidemia [50,61]. In 2 casuistics conducted by Cortes et al. (2011) and Ortiz Ruiz et al. (2016), *C. parapsilosis* (sensu lato) and *C. tropicalis* infected exactly the same percentage of patients [52,55].

Prevalence rates of *C. glabrata* presented a large variation among studies documented in different countries. In general, *C. glabrata* represented the third or fourth most common CNA species documented in *Candida* bloodstream infections, exhibiting prevalence rates ranging from 1% to 13.5%. The highest rate for this species was found by Corzo-Leon et al. (2014) in Mexico where *C. glabrata* was considered the second most common cause of CNA candidemia behind only *C. tropicalis* [58].

Prevalence rates of *C. krusei* strains ranged from 0 to 6% among all studies. *C. guilliermondii* (sensu lato) was isolated in 33 out 40 studies, being reported in almost all countries represented in this review. The prevalence of *C. guilliemondii* candidemia ranged from 0 to 12%. The highest prevalence rate of *C. guilliermondii* candidemia was found in Honduras, as reported by Nucci et al. (2013), along with the characterization of 672 episodes of candidemia carried out in Latin America [7].

All species other than *C. albicans*, *C. parapsilosis* (sensu lato), *C. tropicalis*, *C. glabrata*, *C. krusei,* and *C. guilliermondii* (sensu lato) were classified as other *Candida* species (OCSs). The highest rate of OCSs among the studies retrieved was observed by Doi et al. (2016) in Brazil with 13.9% of its collection [51]. Overall, the most common species documented, in at least 5 out of 40 studies, were *C. rugosa*, *C. pelliculosa*, *C. lusitaniae*, *C. famata*, *C. lipolytica*, and *C. kefyr*. The OCSs less frequently isolated were *C. haemulonii*, *C. intermedia*, *C. sake*, *C. holmii*, *C. zeylanoides*, *C. utilis*, *C. viswanathii*, *C. dubliniensis*, and *C. novergensis.*

Overall, we were able to identify historical trends in species distribution of *Candida* spp. only related to prevalence of *C. glabrata*. In this regard, as illustrated on Table 1, only 1 out of 8 Brazilian studies published between 1997 and 2006 presented prevalence rates of *C. glabrata* candidemia ≥7%. Otherwise, 7 out of 14 studies published between 2007 and 2016 exhibited prevalence rates ≥7%. Similarly, in Argentina there was a substantially increment of *C. glabrata* rates moving from a rate of 2.7% in the first study reported in 2005 to 10% in a recent casuistic involving 158 episodes of candidemia reported by Riera et al. (2014) [31]. In Colombia, prevalence rates of *C. glabrata* in candidemia remained lower than 3% along the first 3 studies, as illustrated on Figure 1, moving up to 6% in a recent study involving 81 episodes of fungemia.

It is seems that there was a slight increase in the prevalence rate of *C. krusei* candidemia in Brazil. Indeed, 7 out of 8 studies from Brazil exhibited prevalence rates of *C. krusei* ≤ 2% between 1997 and 2006 [32,33,35,36,38,39,64]. In contrast, 5 out of 14 studies published between 2007 and 2016 exhibited isolation rates ≥2% for this particular species [15,42,43,45,48]. Prevalence rates of *C. krusei* candidemia remained stable along different studies reported in Argentina and Colombia (see Table 1).

### Temporal Trends of Antifungal Resistance to Azoles and Echinocandins

An overall review of the 40 studies retrieved for *Candida* species distribution analysis, only 17 of them presented reliable information on the antifungal susceptibility tests of strains using broth microdilution methods standardized by CLSI and EUCAST. In order to check for trends in terms of rising antifungal resistance along the study period, we compared data from 7 studies published between 1997 and 2006 to data generated from 10 studies published between 2007 and 2016 (Table 2).

Ten out of the 17 studies providing data on *Candida* antifungal resistance were performed in Brazilian medical centers, 2 were from Argentina, 2 from Mexico, and 1 from Peru. We included an analysis of data generated by two multicenter studies that provided additional information from Argentina, Brazil, Chile, Colombia, Ecuador, Honduras, Peru, and Venezuela.

Reviewing the antifungal drugs that were evaluated along the 17 studies, we found that only fluconazole was tested in all them, followed by amphotericin B in 15 studies. Other drugs frequently tested included voriconazole (11 studies), caspofungin (5 studies), and anidulafungin (4 studies). Data on micafungin was only reported in one study.

It is important to mention that no single author from the Latin American surveys reporting rates of antifungal resistance of *Candida* bloodstream isolates, either to triazoles or echinocandins, attempted to confirm their in vitro profile by checking the expression of molecular mechanisms of antifungal resistance.

(i) Trends in resistance to fluconazole and voriconazole among *C. albicans*, *C. tropicalis,* and *C. parapsilosis* isolates.

Resistance to azoles among isolates of *C. albicans*, *C. tropicalis*, and *C. parapsilosis* remained relatively unchanged over time. Except for one single study conducted by Rodero et al. (2005) in Argentina, all studies carried out prior to 2006 showed that less than 1% of isolates exhibited a non-susceptible (susceptible in a dose dependent manner [SDD] or resistant) phenotype to fluconazole and voriconazole. Regarding the Argentinean casuistic, the authors reported fluconazole resistance rates of 15.7% and 1.8% for *C. albicans*, and 43% and 5.4% for *C. tropicalis*, by using CLSI and EUCAST, respectively [28].

After 2007, a slight increase in the percentage of resistance to fluconazole was noted. Overall, resistance rates to fluconazole increased from 0.4% to 1.2% among *C. albicans*, from 0.5% to 2.3% among isolates of *C. tropicalis*, and from 0 to 2.6% for *C. parapsilosis* [7,15,29,46,49,50,57,59]. For voriconazole, resistance rates remained around 1%.

(ii) Trends in resistance to echinocandins among *Candida* spp.

Only 7 out of 17 studies, all published after 2007, have provided data on antifungal susceptibility of at least one of the 3 echinocandins available for clinical use in Latin America. Of note, echinocandin resistance was reported in only 3 out of 7 mentioned studies. In Brazil, Santos et al. (2014) reported that 3 out of 15 (20%) of *C. glabrata* isolates were non-susceptible to caspofungin [49]. Similar data were observed by Bustamante et al. (2014) in Peru where 1 out of 8 (12.5%) of *C. glabrata* strains was resistant to anidulanfungin [59]. The only study reporting in vitro echinocandin resistance against species other than *C. glabrata* was conducted by Cordoba et al. (2011) in Argentina where they found 26 out of 120 (21.6%) of *C. parapsilosis* isolates resistant to anidulafungin by using the EUCAST microdilution method [29].

## 4. Discussion

Candidemia remains a significant public health problem worldwide [1,2,3,4,5,6,7]. In Latin America, the incidence rates range from 0.74 to 6.0 per 1000 hospital admissions [30,38,45,48,56,58]. Despite all advances related to the development of new diagnostic and therapeutic tools for fungal infections, crude mortality rates of candidemia remain high in Latin American medical centers, ranging from 30 to 76% [28,42,44,51,55,56,58,60].

As expected, *C. albicans* remains the most common species causing candidemia in our region, followed by *C. parapsilosis* and *C. tropicalis* [65,66]. Otherwise, prevalence rates of *C. glabrata* were highly variable within different studies and different countries. The same variation observed among several countries was noted in one recent global trend review of *Candida* species distribution, where prevalence rates of *C. glabrata* in candidemic patients in European medical centers ranged between 8% (Spain) and 20% (Denmark) [10]. In Brazil, Argentina and Colombia, we noted a trend towards an increase in *C. glabrata* frequency [28,31,41,48] along the period of study. This increase has an important clinical impact, as *C. glabrata* shows a diminished susceptibility to azoles and, eventually, candins [27]. Although the explanation for this shift is not completely understood, it seems to be a consequence of the increased exposure to fluconazole, as previously demonstrated by several authors [5,13].

*C. krusei* and *C. guilliermondii* (sensu lato) frequencies have not demonstrated substantial changes in their frequency, but both species were found in most studies from different countries. In fact, *C. krusei* was found in 36 out of 40 articles, while *C. guilliermondii* (sensu lato) was found in 33. Of note, in the single multicenter study conducted by Nucci et al. (2013) in 7 countries, *C. guilliermondii* ranged from 1.6% to 20% [7]. Due to their reduced susceptibility to fluconazole, both *C. krusei* and *C. guilliermondii* may have a relevant clinical impact in terms of defining strategy for empirical therapy of risk patients [67,68].

One limitation found in all studies reviewed is that only phenotypic methods were used to identify *Candida* species involved in the episodes of candidemia. Consequently, accurate identification of cryptic species of *Candida* could not be provided. In addition, we may not exclude the possibility that the OCSs reported by the authors, including *C. rugosa*, *C. pelliculosa*, *C. lusitaniae*, *C. famata*, *C. lipolytica*, and *C. kefyr*, were misidentified by inaccurate phenotypic methods used by the routine laboratories. There is a consensus that MALDI-TOF and sequencing of the Internal Transcribed Spacer (*ITS*) region of ribosomal DNA are both considered to be far more accurate and reliable for providing identification of cryptic and rare species of *Candida* than conventional methods [69,70,71].

In this regard, despite the fact that *C. auris* strains have not been detected as OCSs causing infection along the 40 mentioned surveys of candidemia performed in our region, two recent reports documented outbreaks of *C. auris* candidemia in medical centers from Venezuela and Colombia [72,73]. This microorganism has been recognized as a multidrug-resistant yeast pathogen since all *C. auris* strains are fully resistant to fluconazole and part of them may be either resistant to candins and/or amphotericin B. We may not exclude the possibility that the real prevalence of this emerging multidrug-resistant yeast pathogen is underestimated in our region since it has been misidentified in routine laboratories as *C. famata* and *C. haemulonii*, among others [74].

Fluconazole resistance has been increasingly noted in *Candida* spp., not only with *C. glabrata* but also among strains initially described as primarily sensitive to azoles such as *C. parapsilosis* and *C. tropicalis* [8,10,20,21,22,24,75,76,77]. In the review presented, we decided not to focus our analysis on fluconazole resistance against *C. glabrata* strains since fluconazole is not considered a safe alternative anymore for treating candidemic patients infected by this pathogen [67,68].

Several studies have reported variations in fluconazole resistance rates among *C. parapsilosis* and *C. tropicalis* isolates. Indeed, resistance rates among *C. parapsilosis* isolates ranged from 3.4% to 7.5% in USA and from 0 to 6% in Europe [8,20,21,22,75,76,77,78]. For *C. tropicalis*, resistance rates ranged from 2.4% to 11.6% in USA and from 1.7% to 22% in Europe [8,20,21,22,75,76,77,78]. In Latin America, we have noted a slight increase in *C. albicans*, *C. parapsilosis*, and *C. tropicalis* isolates exhibiting a non-susceptible profile to fluconazole along our study period. Indeed, resistance rates to fluconazole increased from 0.4% to 1.2% among *C. albicans* isolates; from 0.5% to 2.3% among isolates of *C. tropicalis* and from 0 to 2.6% for *C. parapsilosis* strains. Of note, though still considered rare, a recent publication from Brazil described an outbreak of *C. parapsilosis* (sensu stricto) candidemia involving 23 intensive care unit (ICU) patients from a single institution [24]. Resistance to fluconazole in *C. parapsilosis* strains in this particular report was confirmed by the presence of *ERG11* mutations and overexpression of efflux pumps by the mentioned strains [79].

Resistance to echinocandins has been described as an uncommon phenomenon among *C. albicans*, *C. parapsilosis*, *C. tropicalis*, and *C. krusei* isolates [80]. However, it has become increasingly common among *C. glabrata* strains from patients admitted in US and European hospitals [27,80,81,82]. In Latin America, apparently, echinocandin resistance remains rare. It is important to mention that echinocandins are rarely used for the treatment of candidemic patients in Latin America in spite of the drug’s availability in the early 2000s. 

According to Nucci et al. (2013), only 40 (5.9%) out of 672 patients with candidemia documented between 2008 and 2010 were initially treated with echinocandins [7]. Another aspect to consider is that antifungal resistance in Latin America may be underestimated since most routine laboratories do not perform antifungal susceptibility tests. 

In our analysis, only 3 out of 40 studies documented any rate of echinocandin resistance with *Candida* sp. bloodstream isolates. Santos et al. (2014) and Bustamante et al. (2014) reported resistance rates of 20% and 12.5% to echinocandins among *C. glabrata* isolates, respectively [49,59]. Cordoba et al. (2011) reported that 21.6% of *C. parapsilosis* isolates were resistant to anidulafungin [29]. However, the high rates of resistance reported by these papers should be interpreted very carefully since they have not confirmed the echinocandin resistance phenotype by checking the presence of fks mutations in all isolates exhibiting high values of minimum inhibitory concentration (MIC) for echinocandins.

Currently, only two reports from medical centers in Latin America have demonstrated microbiological and molecular echinocandin resistance against *Candida* bloodstream isolates. Bizerra et al. (2014), using sequencing methodology for the study of *FKS* genes and quantification of glucan synthesis, reported the occurrence of a mutation associated with the resistance phenotype against echinocandins in *C. glabrata* isolated from a single cancer patient with candidemia exposed to antifungal prophylaxis with micafungin [25]. Finally, Forastiero et al. (2015) reported a rapid development of resistance in *C. krusei* isolates recovered from a patient under caspofungin treatment. Clinical resistance was associated with increased echinocandin MICs and was ultimately related to new mutations of the target enzyme (fks1) [83].

## 5. Conclusions

In conclusion, there has been a changing epidemiology of *Candida* bloodstream infection over the past years in Latin America. Although *C. albicans* remains the predominant cause of candidemia, a shift has been reported in the epidemiology as some CNA species have emerged as the cause of candidemia, and they can exhibit resistance to fluconazole and echinocandins. Several medical centers have reported episodes of candidemia due to *C. tropicalis* and *C. parapsilosis* isolates resistant to fluconazole. Echinocandin resistance confirmed by molecular studies has been reported in only two episodes of candidemia reported in patients assisted at medical centers from Latin America, and appears to be rare in our region. Continuous multicenter surveillance studies of candidemia in Latin America are necessary to detect any regional and historical trends, in terms of *Candida* species distribution and emergence of antifungal resistance, early. This information is critical to clinicians in preventing and controlling *Candida* bloodstream infections in our medical centers.

## Figures and Tables

**Table 1:** Species distribution of *Candida* bloodstream isolates from Latin American and Caribbean medical centers (1997–2016).

Country/Territory	Reference	Period	n ^e^ (No. of Centers)	Species Distribution (%)						
				*Ca* ^a^	*Cp* ^a^	*Ct* ^a^	*Cgla* ^a^	*Ck* ^a^	*C. gui* ^a^	OCS ^b^
Argentina	Rodero et al. (2005) [28] ^c^	1999–2000	253 (36)	42.5	29.9	16.5	2.7	1.18	0.39	6.83
Argentina	Cordoba et al. (2011) [29] ^c^	2007–2008	420 (47)	42.1	28.5	16.9	4.7	0.47	1.66	5.67
Argentina	Lopez Moral et al. (2012) [30] ^c^	2005–2008	683 (16)	41.3	24.3	19.9	6.3	0.59	1.76	5.85
Argentina	Riera et al. (2014) [31]	2010–2012	158 (4)	41	20	12	10	1.5	3	12.5
Brazil	Colombo et al. (1999) [32]	1995–1996	145 (6)	37	25	24	4	1	2	7
Brazil	Costa et al. (2000) [33] ^c^	1994–1996	84 (1)	51.2	17.8	11.9	2.3	1.19	9.5	6.11
Brazil	Colombo et al. (2003) [34]	1996–1998	200 (5)	41.5	20.5	24	4.5	1	2	6.5
Brazil	Goldani and Mario (2003) [35] ^c^	1996–1999	101 (1)	32.6	23.7	26.7	8.9	1.9	0	6.2
Brazil	Antunes et al. (2004) [36]	2002–2003	120 (1)	48.3	25.8	13.3	3.3	1.7	1.6	6
Brazil	Aquino et al. (2005) [37]	1998–2004	131 (1)	45	24.4	15.3	6.9	4.6	0.76	3.04
Brazil	Colombo et al. (2006) [38]	2003–2004	712 (11)	40.9	20.5	20.9	4.9	1.1	2.4	9.3
Brazil	Medrano et al. (2006) [39]	2000–2002	50 (1)	28	36	16	4	0	12	4
Brazil	Colombo et al. (2007) [40]	2002–2003	282 (4)	38	23	17	3	1	6	12
Brazil	da Matta et al. (2007) [41]	1995–2003	1000 (4)	40	23.8	24.3	4.4	0.6	3	3.9
Brazil	Girao et al. (2008) [42]	1999–2006	108 (1)	40	18	34	1	3	1	3
Brazil	Franca et al. (2008) [43]	2001–2004	100 (1)	59	9	15	7	2	2	6
Brazil	Motta et al. (2010) [44]	2006	136 (1)	52.2	22.1	14.7	6.6	1.6	0.8	2
Brazil	Sampaio Camargo et al. 2010 [45]	1997–2007	151 (1)	44	22	15	9	6	0.66	3.34
Brazil	Bonfietti et al. (2012) [46]	1998–2006	100 (1)	44	37	13	5	1	0	0
Brazil	Hoffmann-Santos et al. (2013) [47]	2006–2011	130 (2)	34.6	38.4	18.4	5.3	0	1.5	1.8
Brazil	Moretti et al. (2013) [48]	2006–2010	313 (1)	44	14.4	21.7	11.2	3.5	1.3	3.9
Brazil	Colombo et al. (2013) [15]	2006–2007	300 (9)	34	26	24	7	3	2	4
Brazil	Santos et al. (2014) [49]	1995–2009	422 (1)	35.7	46.6	9.7	3.5	0.94	1.65	1.91
Brazil	Colombo et al. (2014) [2]	2003–2012	1392 (22)	42	19	20	9	1.14	0.86	8
Brazil	da Costa et al. (2014) [50]	2006–2011	108 (1)	28.7	24.1	30.5	8.3	1.8	0	6.6
Brazil	Doi et al. (2016) [51]	2007–2010	137 (16)	34.3	24.1	15.3	10.2	1.5	0.7	13.9
Colombia	Cortes et al. (2011) [52] ^c^	2001–2007	1622 (27)	50.9	15.5	15.5	2	2.36	3.3	10.44
Colombia	Cortes et al. (2013) [53]	2004–2008	382 (7)	56	16	17.3	2.6	0.8	0	7.3
Colombia	Cortes et al. (2014) [54]	2008–2009	131 (7)	66.4	14.5	10.6	1.5	0	1.5	5.5
Colombia	Ortiz Ruiz et al. (2016) [55]	2008–2012	81 (3)	52	17	17	6	3	4	1
Costa Rica	Villalobos et al. (2016) [56]	2007–2011	210 (1)	38	42	10	7	NR ^f^	NR	3
Mexico	Gonzalez et al. (2008) [57]	2004–2007	398 (5)	31.9	37.9	14.8	8	2.7	1.3	3.3
Mexico	Corzo-Leon et al. (2014) [58]	2008–2010	74 (2)	46	5	26	13.5	5	3	1.5
Peru	Bustamante et al. (2014) [59]	2009–2011	153 (1)	39.9	28.1	23.5	5.2	0.7	2	0.6
Puerto Rico	Conde-Rosa et al. (2010) [60]	2005–2006	85 (1)	28	49	17	4	1	0	1
Venezuela	Franco et al. (2008) [61]	2003–2005	154 (6)	18.8	26	39	7.8	2.6	0	5.8
Multic ^d^	Pfaller et al. (2000) [62]	1997–1998	107 (7)	43	26.1	16.8	6.6	0.9	0	6.6
Multic	Pfaller et al. (2001) [63]	1997–1999	132 (9)	45	25	16	6	1	NR	7
Multic	Godoy et al. (2003) [64]	1999–2000	103 (5)	42	21.3	24.2	7.7	0	2.9	1.9
Multic	Nucci et al. (2013) [7]	2008–2010	672 (21)	37.6	26.5	17.6	6.3	2.7	6.5	2.8

^a^ *Ca*: *C. albicans*. *Cp*: *C. parapsilosis* (sensu lato). *Ct*: *C. tropicalis*. *Cgla*: *C. glabrata*. *Ck*: *C. krusei*. *Cgui*: *C. guilliermondii* (sensu lato); ^b^ Other Candida Species—species other than *C. albicans*. *C. parapsilosis* (sensu lato). *C. tropicalis*. *C. glabrata*. *C. krusei*. *C. guilliermondii* (sensu lato); ^c^ Fungemia collection. Percentage of species distribution was recalculated taking in account only Candida species as denominator; ^d^ Multicenter studies including different countries in Latin America; ^e^ Number of Candida isolates; and ^f^ NR: Not Reported.

**Table 2:** Azole resistance rates of *C. albicans*, *C. parapsilosis* (sensu lato) and *C. tropicalis* bloodstream isolates from Latin American medical centers (1997–2016).

Country	Reference; Number of Isolates	Method	Species	Fluconazole		Voriconazole	
				SDD ^a^	R ^b^	SDD	R
Argentina	Rodero et al. (2006) [28];	CLSI ^c^ ×	*Ca* ^e^	NR ^i^	15.7% (1.8%)	NT ^j^	NT
	*n* = 265	(EUCAST)	*Cp* ^f^	NR	0% (0%)	NT	NT
			*Ct* ^g^	NR	43% (5.4%)	NT	NT
Argentina	Cordoba et al. (2011) [29];	EUCAST ^d^	*Ca*	NR	0%	NR	0.5%
	*n* = 420		*Cp*	NR	2.5%	NR	0.8%
			*Ct*	NR	4.2%	NR	4.2%
Brazil	Colombo et al. (2003) [34];	CLSI	*Ca*	1.2%	0%	NT	NT
	*n* = 200		*Cp*	0%	0%	NT	NT
			*Ct*	0%	0%	NT	NT
Brazil	Antunes et al. (2004) [36];	CLSI	*Ca*	0%	0%	NT	NT
	*n* = 120		*Cp*	0%	0%	NT	NT
			*Ct*	0%	0%	NT	NT
Brazil	Aquino et al. (2005) [37];	CLSI	*Ca*	0%	0%	NT	NT
	*n* = 131		*Cp*	0%	0%	NT	NT
			*Ct*	0%	0%	NT	NT
Brazil	Colombo et al. (2006) [38];	CLSI	*Ca*	0.3%	0.3%	0%	0.3%
	*n* = 712		*Cp*	0%	0%	0%	0%
			*Ct*	1.3%	0%	0%	0%
Brazil	Colombo et al. (2007) [40];	CLSI	*Ca*	0%	0%	NT	NT
	*n* = 282		*Cp*	0%	0%	NT	NT
			*Ct*	0%	0%	NT	NT
Brazil	da Matta et al. (2007) [41];	CLSI	*Ca*	0%	0%	0%	0%
	*n* = 1000		*Cp*	0%	0%	0%	0%
			*Ct*	0%	0%	0%	0%
Brazil	Bonfietti et al. (2012) [46];	EUCAST	*Ca*	0%	0%	0%	0%
	*n* = 100		*Cp*	6%	0%	0%	3%
			*Ct*	0%	0%	0%	0%
Brazil	Colombo et al. (2013) [15];	CLSI	*Ca*	0%	0%	0%	0%
	*n* = 300		*Cp*	1.3%	0%	0%	0%
			*Ct*	2.5%	0%	0%	0%
Brazil	Santos et al. (2014) [49];	CLSI	*Ca*	9.9%	0%	2.6%	0%
	*n* = 422		*Cp*	7%	0%	3.5%	0%
			*Ct*	19.5%	7.3%	12%	4.9%
Brazil	da Costa et al. (2014) [50];	EUCAST	*Ca*	NR	3.7%	NR	3.7%
	*n* = 108		*Cp*	NR	26.9%	NR	0%
			*Ct*	NR	3,2%	NR	3.2%
Mexico	Gonzalez et al. (2014) [57];	CLSI	*Ca*	0%	0.8%	0%	0.8
	*n* = 398		*Cp*	0%	0%	0%	0%
			*Ct*	0%	0%	0%	0%
Mexico	Corzo-Leon et al. (2014) [58];	CLSI	*Ca*	0%	0%	0%	0%
	*n* = 74		*Cp*	0%	0%	0%	0%
			*Ct*	0%	0%	0%	0%
Peru	Bustamante et al. (2014) [59];	CLSI	*Ca*	NR	NR	0%	5%
	*n* = 153		*Cp*	NR	2.3%	NR	NR
			*Ct*	NR	NR	NR	NR
Multicenter	Godoy et al. (2003) [64];	CLSI	*Ca*	0%	0%	NT	NT
studies ^h^	*n* = 103		*Cp*	0%	0%	NT	NT
			*Ct*	0%	0%	NT	NT
Multicenter	Nucci et al. (2013) [7];	CLSI	*Ca*	0,4%	0%	0%	0%
studies	*n* = 672		*Cp*	1,1%	0%	0%	0%
			*Ct*	0%	0%	0%	0%

^a^ Susceptible in a dose-dependent manner; ^b^ Resistant to azoles; ^c^ Clinical Laboratory Standard Institute; ^d^ European Committee on Antimicrobial Susceptibility Testing; ^e^ *C. albicans*; ^f^ *C. parapsilosis* (sensu lato); ^g^ *C. tropicalis*; ^h^ Multicenter studies including different countries from Latin America; ^i^ NR: Not reported; ^j^ NT: Not tested.

## Supplementary Materials

The following are available online at www.mdpi.com/2309-608X/3/2/24/s1.
