# Supplementary material for: Revisiting Species Distribution and Antifungal Susceptibility of Candida Bloodstream Isolates from Latin American Medical Centers
Source: J Fungi (Basel). 2017 May 17;3(2):24. doi: 10.3390/jof3020024 (PMC5715916; doi:10.3390/jof3020024)
Supplement: Supplementary file 1 [file jof-03-00024-s001.docx]

**Revisiting species distribution and antifungal susceptibility of *Candida* bloodstream isolates from Latin American medical centers**

**Daniel Archimedes da Matta^1^, Ana Carolina Remondi Souza^1^, Arnaldo Lopes Colombo^1*^**

^1^ Laboratório Especial de Micologia, Disciplina de Infectologia, Escola Paulista de Medicina, Universidade Federal de São Paulo, São Paulo, SP, Brazil; darchimedes@hotmail.com

^2^ Laboratório Especial de Micologia, Disciplina de Infectologia, Escola Paulista de Medicina, Universidade Federal de São Paulo, São Paulo, SP, Brazil; carolina.remondi@yahoo.com.br

^3^ Laboratório Especial de Micologia, Disciplina de Infectologia, Escola Paulista de Medicina, Universidade Federal de São Paulo, São Paulo, SP, Brazil; arnaldolcolombo@gmail.com

***** Correspondence: arnaldolcolombo@gmail.com; Tel.: +55-11-5576-4985

Academic Editor: name

Received: date; Accepted: date; Published: date

**Supplementary Material**

**
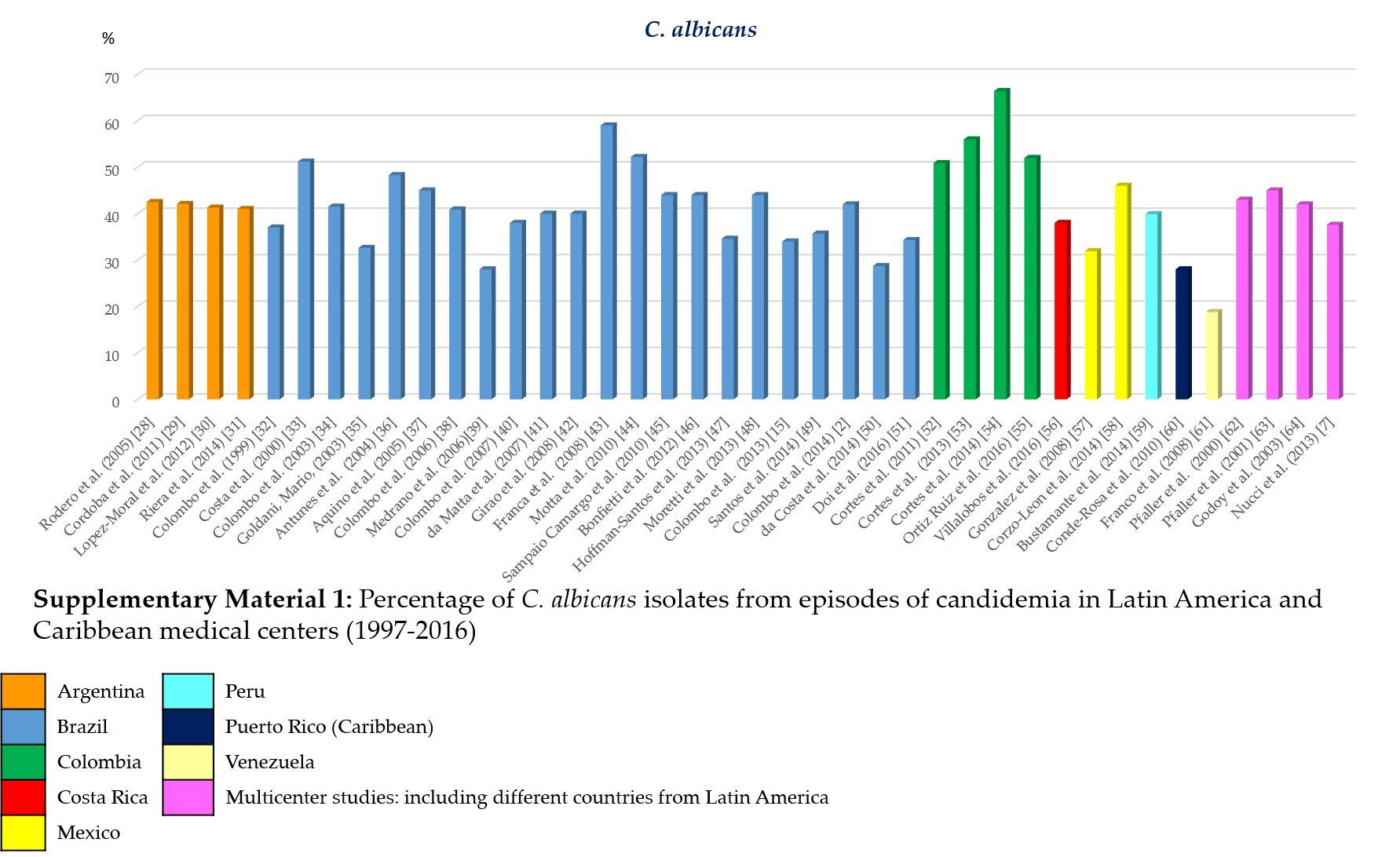

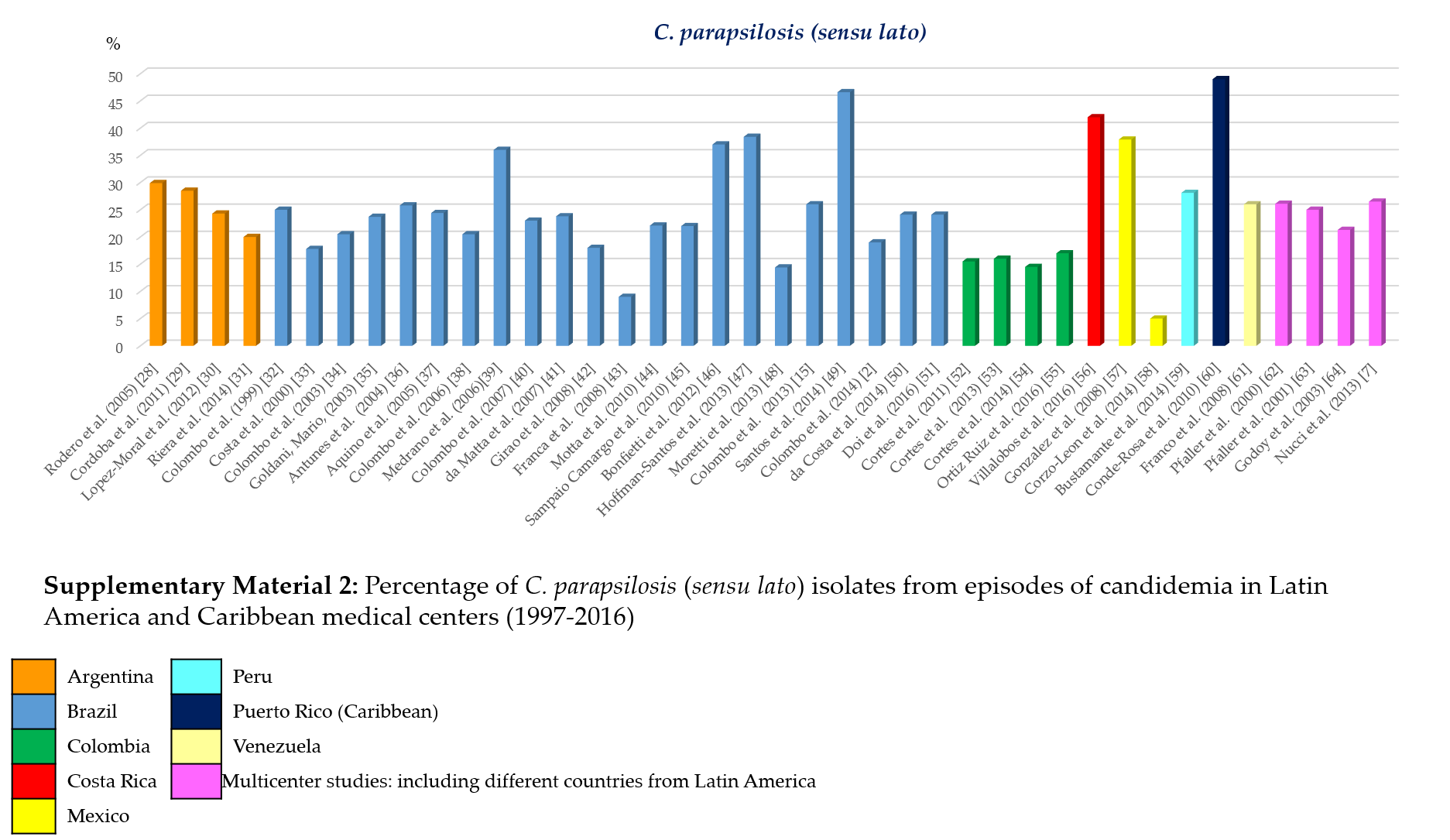

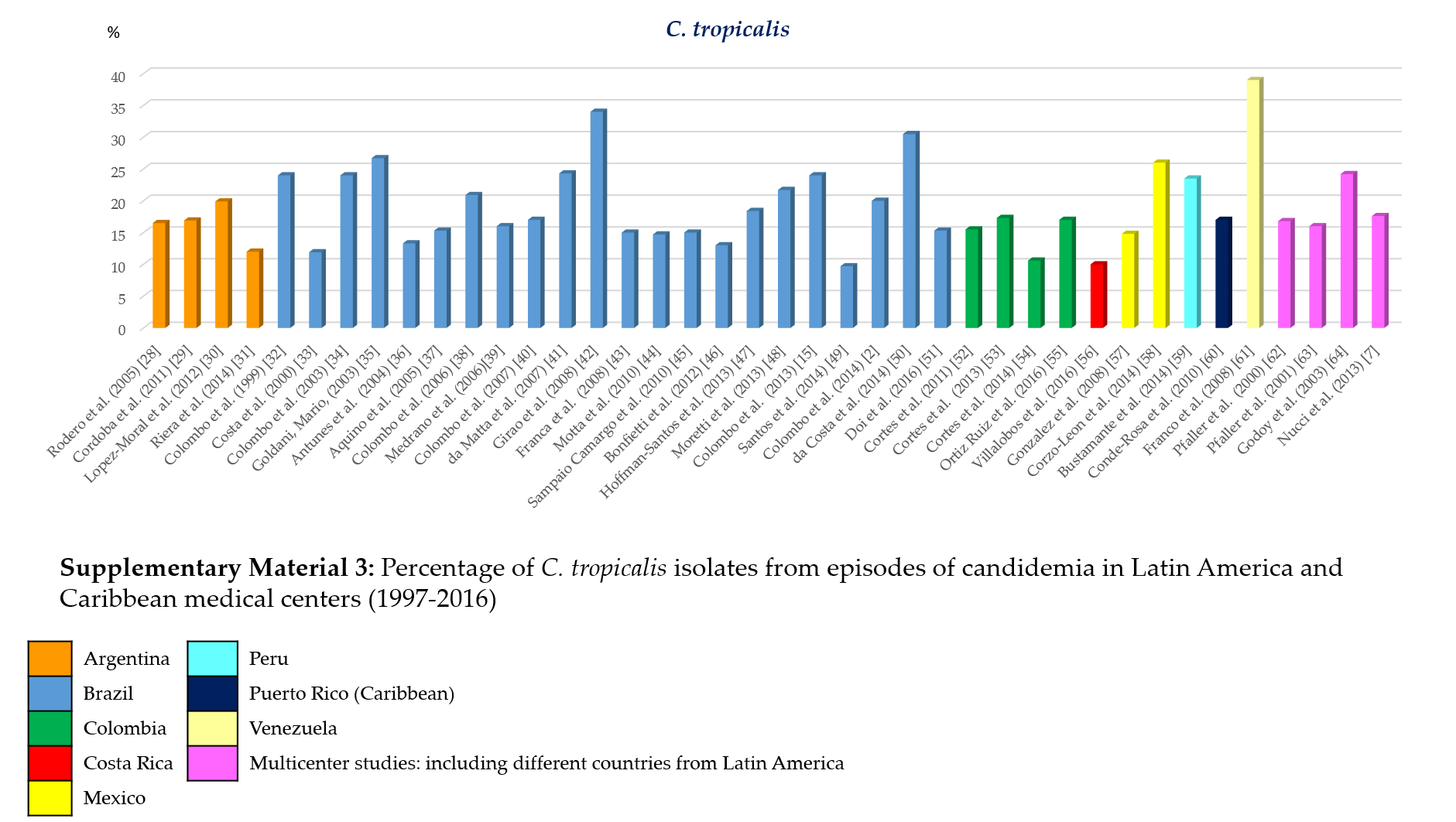

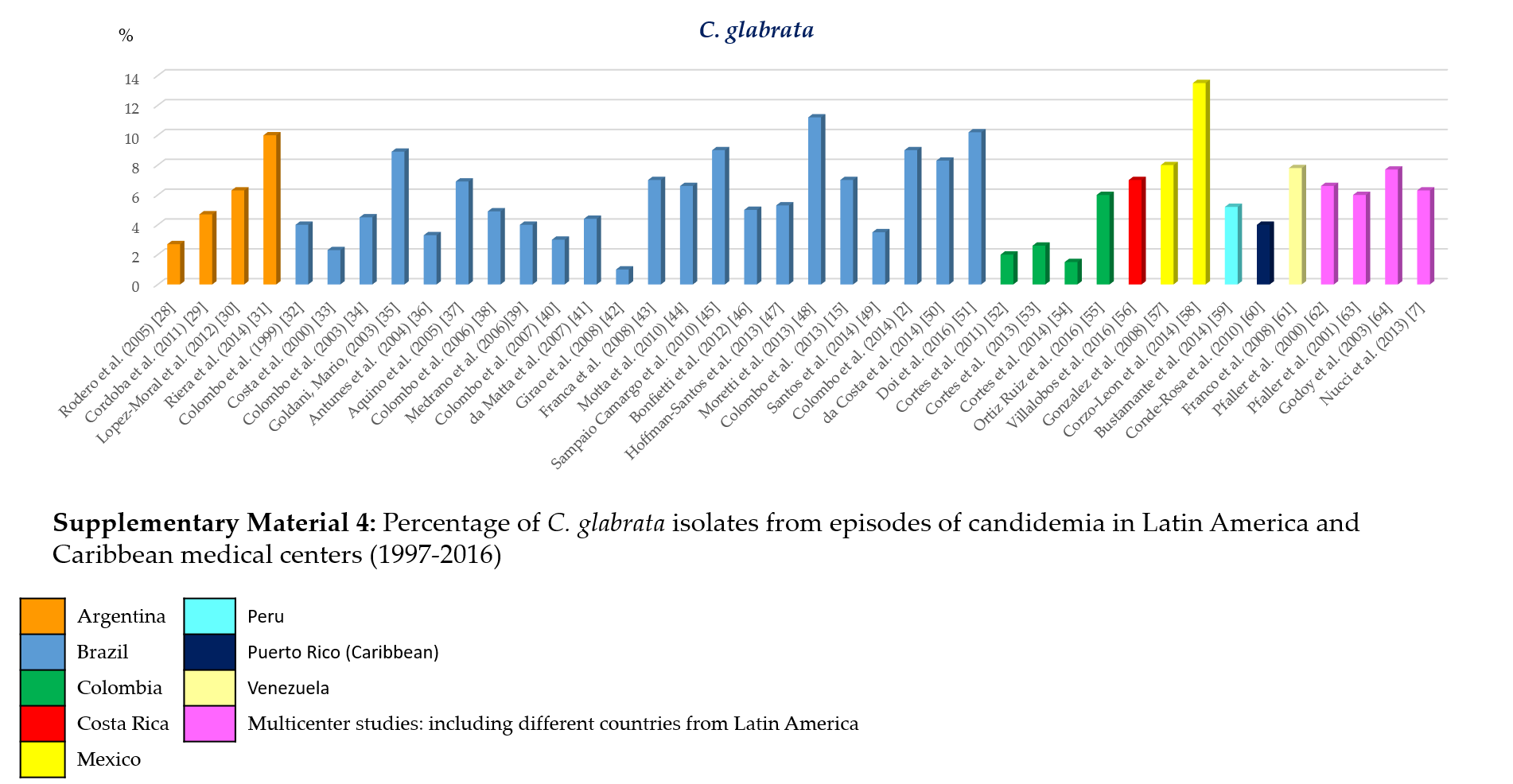

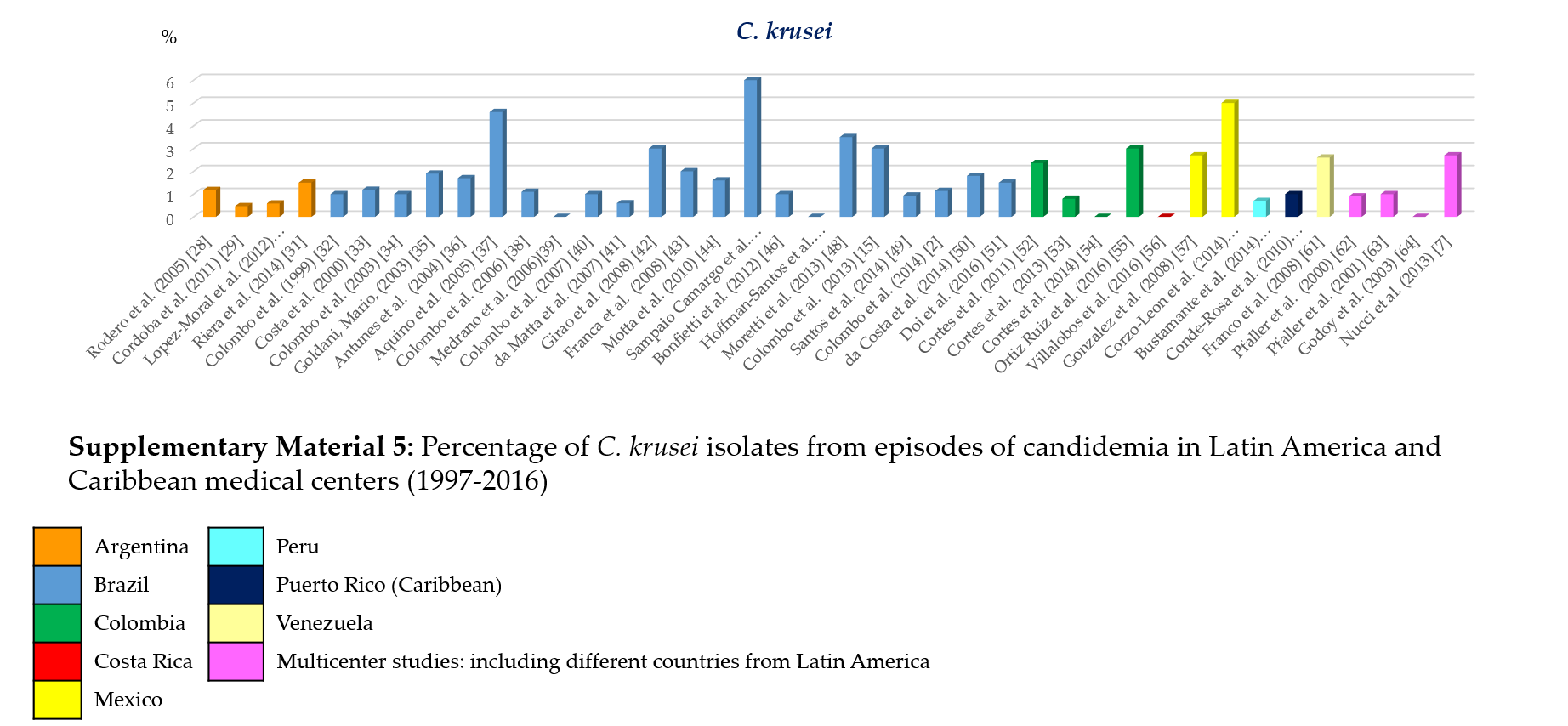

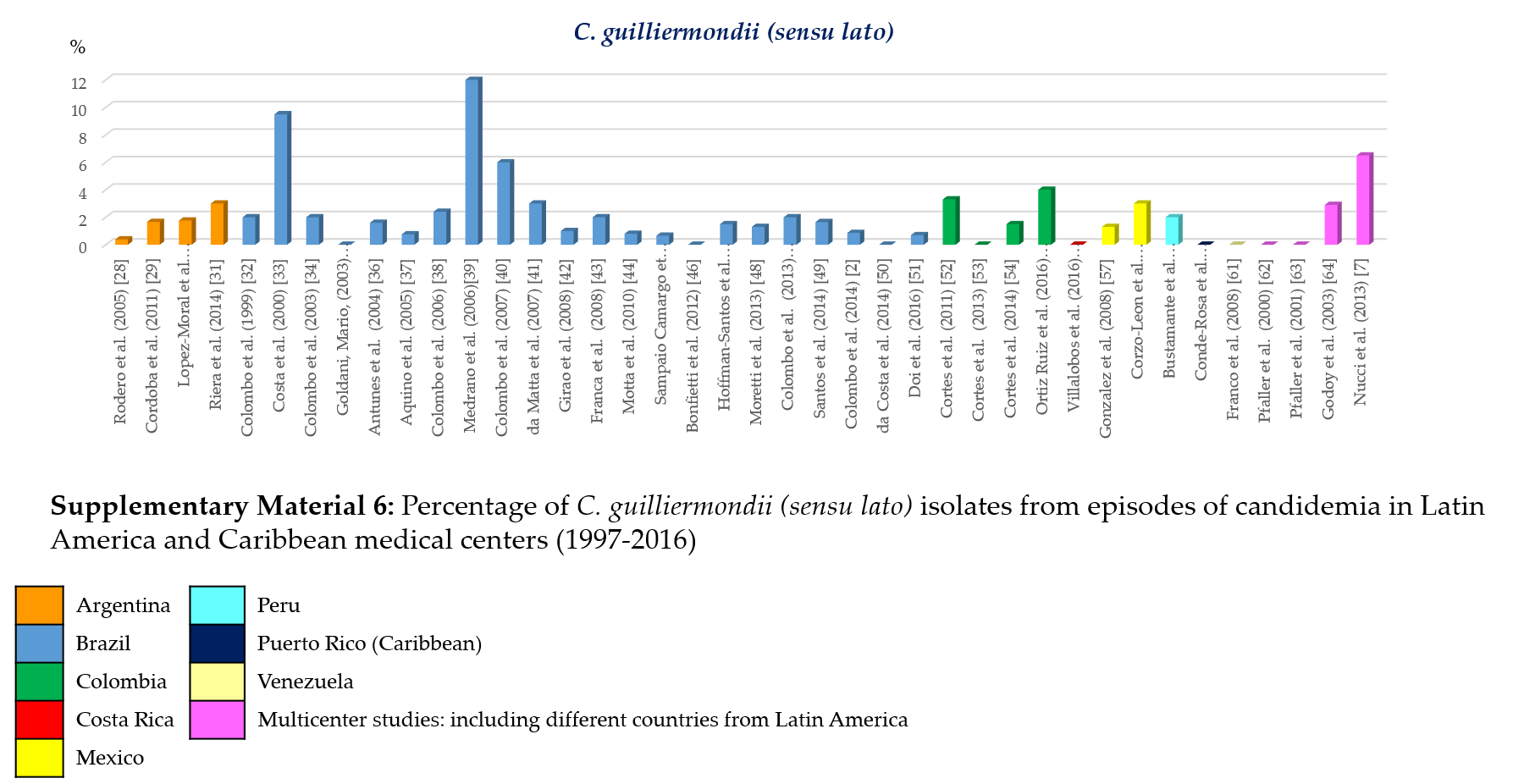

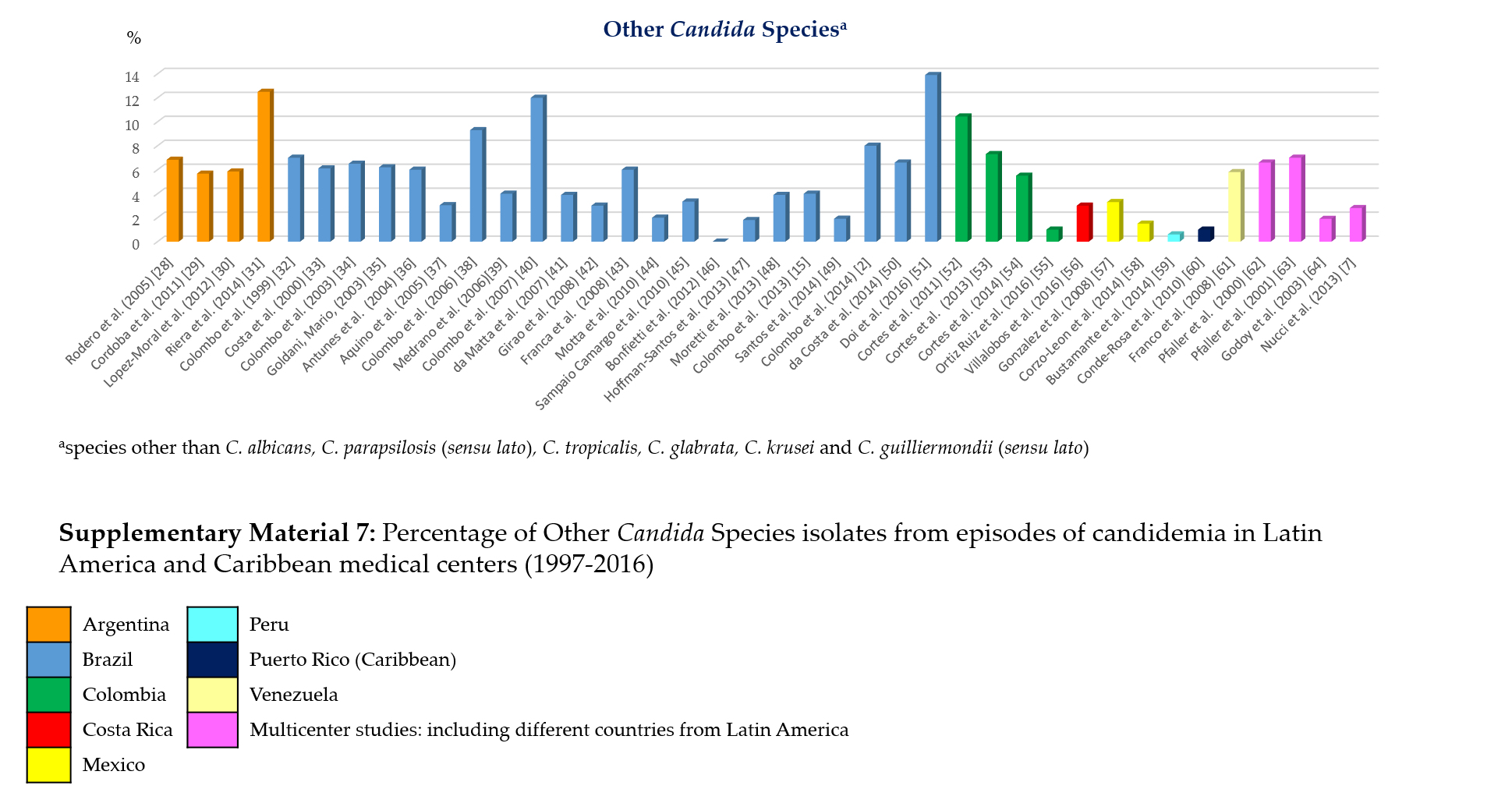
**
